# International clones of extended‐spectrum β‐lactamase (CTX‐M)‐producing *Escherichia coli* in peri‐urban wild animals, Brazil

**DOI:** 10.1111/tbed.13558

**Published:** 2020-04-21

**Authors:** Marcelo P. N. de Carvalho, Miriam R. Fernandes, Fábio P. Sellera, Ralf Lopes, Daniel F. Monte, Alícia G. Hippólito, Liliane Milanelo, Tânia F. Raso, Nilton Lincopan

**Affiliations:** ^1^ Departament of Veterinary Clinics and Surgery Federal University of Minas Gerais Belo Horizonte Brazil; ^2^ Department of Clinical and Toxicological Analysis School of Pharmaceutical Sciences University of Sao Paulo Sao Paulo Brazil; ^3^ Department of Internal Medicine School of Veterinary Medicine and Animal Science University of São Paulo São Paulo Brazil; ^4^ Department of Microbiology Instituto de Ciências Biomédicas Universidade de São Paulo São Paulo Brazil; ^5^ Department of Food and Experimental Nutrition Faculty of Pharmaceutical Sciences Food Research Center University of São Paulo São Paulo Brazil; ^6^ Department of Veterinary Surgery and Anesthesiology School of Veterinary Medicine and Animal Science Universidade Estadual Paulista (UNESP) Botucatu Brazil; ^7^ Reception Center for Wildlife Ecological Park Tietê São Paulo Brazil; ^8^ Department of Pathology School of Veterinary Medicine and Animal Science University of São Paulo São Paulo Brazil

**Keywords:** Enterobacterales, ESBL, MDR bacteria, resistome, wildlife

## Abstract

CTX‐M‐type extended‐spectrum β‐lactamase (ESBL)‐producing *Escherichia coli* clones have been increasingly reported worldwide. In this regard, although discussions of transmission routes of these bacteria are in evidence, molecular data are lacking to elucidate the epidemiological impacts of ESBL producers in wild animals. In this study, we have screened 90 wild animals living in a surrounding area of São Paulo, the largest metropolitan city in South America, to monitor the presence of multidrug‐resistant (MDR) Gram‐negative bacteria. Using a genomic approach, we have analysed eight ceftriaxone‐resistant *E. coli*. Resistome analyses revealed that all *E. coli* strains carried *bla*_CTX‐M_‐type genes, prevalent in human infections, besides other clinically relevant resistance genes to aminoglycosides, β‐lactams, phenicols, tetracyclines, sulphonamides*,* trimethoprim, fosfomycin and quinolones. Additionally, *E. coli* strains belonged to international sequence types (STs) ST38, ST58, ST212, ST744, ST1158 and ST1251, and carried several virulence‐associated genes. Our findings suggest spread and adaptation of international clones of CTX‐M‐producing *E. coli* beyond urban settings, including wildlife from shared environments.

## 1 INTRODUCTION

The spread of extended‐spectrum β‐lactamase (ESBL)‐producing Enterobacterales has been broadly reported worldwide (Brolund, 2014; Fernandes et al., 2018; Pardon et al., 2015). In this respect, a number of interlinked factors, such as food animals, environmental sources, human migration and access to basic sanitation in highly populated cities, are contributing for the accelerated dissemination of these bacteria in urban and wild environments (Radhouani et al., 2014; Sacramento et al., 2018; Sellera, Fernandes, Moura, Carvalho, & Lincopan, 2018).

While the exposure to polluted environments constitutes a risk factor for humans to acquire multidrug‐resistant (MDR) bacteria, recent studies have pointed out that it could also have implications for wildlife (Cerdà‐Cuéllar et al., 2019; Sellera, 2019; Wang et al., 2017). In fact, although this matter remains poorly addressed under ecological perspectives, the scientific community and nature conservation authorities have begun to see wild animals as reservoirs and potential disseminators of ESBL‐producing bacteria (Ardiles‐Villegas, González‐Acuña, Waldenström, Olsen, & Hernández, 2011; Cerdà‐Cuéllar et al., 2019; Sellera, 2019; Wang et al., 2017). Nowadays, most ESBL‐producing *Escherichia coli* circulating at the human–animal–environment interface belong to international sequence types (STs) such as ST10, ST38, ST58, ST131, ST212, ST648, ST744, ST1158 and ST1251 (Borges, Tarlton, & Riley, 2019; Cao et al., 2014; Castellanos et al., 2017; Haenni et al., 2018; Nüesch‐Inderbinen et al., 2019; Pitout, 2012; Tacão et al., 2017; Tafoukt, Touati, Leangapichart, Bakour, & Rolain, 2017; Vignoli et al., 2016; Zurfluh et al., 2017), suggesting a broad host adaptation of these pathogens. In this study, we report the occurrence of pandemic clones of CTX‐M‐producing *E. coli* recovered from a diversity of peri‐urban wild animals in Brazil, highlighting the transmission of this sort of bacteria in anthropogenic‐shared environments.

## 2 MATERIALS AND METHODS

Between June 2017 and July 2018, a local surveillance study was conducted to monitor the presence of MDR Gram‐negative bacteria in urbanized wild animals, in São Paulo, Brazil, the largest metropolitan city in South America. For this purpose, we sampled rectal or cloacal swabs from 90 wild animals, including reptiles, birds and mammals’ species rescued by authorities (firefighters and environmental police) and delivered to wildlife rehabilitation centres. The sampled species included *Alouatta guariba* (*n* = 4), *Asio clamator* (*n* = 7), *Asio stygius* (*n* = 1), *Caracara plancus* (*n* = 2), *Coragyps atratus* (*n* = 27), *Didelphis aurita* (*n* = 11), *Egretta thula* (*n* = 1), *Hydrochoerus hydrochaeris* (*n* = 14), *Hydromedusa tectifera* (*n* = 2), *Megascops choliba* (*n* = 2), *Nasua nasua* (*n* = 13), *Nycticorax nycticorax* (*n* = 1), *Sapajus apellla* (*n* = 1), *Tapirus terrestris* (*n* = 1), *Tupinambis merianae* (*n* = 2) and *Tyto furcata* (*n* = 1). Biological sample collections were authorized by the Authorization System and Information on Biodiversity (SISBIO licence number 55804–2).

Swab samples were streaked onto MacConkey agar plates supplemented with ceftriaxone (2 µg/ml), colistin (2 µg/ml) or meropenem (2 µg/ml), and the grown bacteria were identified by matrix‐assisted laser desorption/ionization time‐of‐flight mass spectrometry (MALDI‐TOF). Antimicrobial susceptibility was determined by disc diffusion and/or E‐test methods, using breakpoints approved by the Clinical and Laboratory Standards Institute (CLSI, 2015, 2017). Twenty‐two antibiotics were tested including amikacin, amoxicillin/clavulanic acid, ampicillin, aztreonam, ceftazidime, cephalothin, ciprofloxacin, chloramphenicol, ceftriaxone, ceftiofur, cefotaxime, doxycycline, enrofloxacin, cefepime, gentamicin, nalidixic acid, sulphonamide, trimethoprim/sulphamethoxazole, tetracycline, kanamycin, tobramycin and streptomycin. Additionally, the presence of CTX‐M‐type (*bla*_CTX‐M‐1,_ *bla*_CTX‐M‐2,_ *bla*_CTX‐M‐8_ and *bla*_CTX‐M‐9_) groups, carbapenemase (*bla*_KPC‐2_) and mobilized colistin resistance (*mcr‐1*) genes was evaluated by PCR analysis (Dropa et al., 2016; Liu et al., 2016; Minarini, Poirel, Trevisani, Darini, & Nordmann, 2009; Muzaheed et al., 2008; Poirel, Walsh, Cuvillier, & Nordmann, 2011; Saladin et al., 2002).

The isolates confirmed positive by PCR were whole‐genome sequenced. Genomic DNA was extracted from overnight cultures using the PureLink^®^ Genomic DNA Mini Kit (Thermo Fisher Scientific) according to the manufacturer's instructions. Whole‐genome sequencing (WGS) was performed using Illumina NextSeq500 platform (Illumina, San Diego, CA) (150 bp paired‐end), and the reads were de novo assembled using Velvet 1.2.10 (Zerbino & Birney, 2008) or SPAdes 3.9 (Bankevich et al., 2012). Sequence types, serotypes, plasmid replicon types, antimicrobial resistance genes and virulence genes were identified using MLST 2.0, SerotypeFinder 2.0 (identity ≥ 85%; coverage ≥ 60%), PlasmidFinder 2.1 (identity ≥ 95%; coverage ≥ 60%), ResFinder 3.2 (identity ≥ 90%; coverage ≥ 60%) and VirulenceFinder 2.0 (identity ≥ 90%; coverage ≥ 60%) tools, respectively, available from the Center for Genomic Epidemiology (http://genomicepidemiology.org/). Analysis of the genetic context of *bla*_CTX‐M_ genes was performed with BLASTn and ISFinder analyses (Siguier, Perochon, Lestrade, Mahillon, & Chandler, 2006) followed by manual curation using Geneious 10.2.6.

Plasmid transfer was performed by conjugation using streptomycin‐resistant *E. coli* C600 or azide‐resistant *E. coli* J53 recipient strains in LB broth assays, ratio 3:1 (recipient:donor). Transconjugants were selected using MacConkey agar supplemented with ceftriaxone (2 µg/ml) and streptomycin (2000 µg/ml), or ceftriaxone (2 µg/ml) and sodium azide (200 µg/ml). In transformation assays, plasmids were extracted by the alkaline lysis method (Sambrook & Russel, 2001), and ultra‐competent *E. coli* TOP10 was heat shock transformed as previously described (Inoue, Nojima, & Okayama, 1990), increasing the thermal shock time at 42°C to 1.5 min. Transformants were selected using MacConkey agar supplemented with ceftriaxone (2 µg/ml). Positive transconjugants and transformants strains were confirmed by *bla*_CTX‐M_ genes using PCR.

## 3 RESULTS AND DISCUSSION

In this study, eight ceftriaxone‐resistant *E. coli* isolates (8/90; 8.88%) were recovered from five birds (one owl and four vultures) and three mammals (coatis). MDR profiles, defined as resistant to three or more classes of antibiotics (Magiorakos et al., 2012), were evidenced in six isolates (ECPET11, ECPET31, ECPET36, ICBUR6, ICBUR15 and ICBUR20). ECPET3 displayed resistance only to cephalosporins and aztreonam, whereas ECPET13 was resistant to cephalosporins, aztreonam and nalidixic acid. Additionally, ESBL production was confirmed by double‐disc synergy test (DDST), and PCR analysis revealed the presence of *bla*_CTX‐M_‐type genes in all eight bacterial isolates (Table 1). No MCR‐1‐positive or carbapenemase‐producing bacteria were identified.

**TABLE 1:** Phenotypic and genotypic features of ESBL‐producing *E. coli* strains isolated from peri‐urban wild animals, Brazil

ID strains	Animal sources	ST/CC	Serotype	Virulence genes	Resistance phenotype	Resistance genotype	Plasmid type	Accession number
ECPET3	Black Vulture (*Coragyps atratus*)	212/−	O18/O18ac:H49	*gad*, *iss*, *lpfA*	CRO, CTX, CAZ, CPM, ATM	*bla*_CTX‐M‐55_, *bla*_TEM‐1B_, *mdf(A)*	FII, N	PQET00000000
ECPET11	South American Coati (*Nasua nasua*)	744/10	O89/O162:H10	*gad*, *iss*, *cma*, *iroN*	CRO, CTX, CAZ, CPM, ATM, CIP, NAL, GEN, KAN, TOB, STR, CLO, SUT, TET	*bla*_CTX‐M‐55_, *bla*_TEM‐1B_, *aadA1*, *aadA2*, *aadA5*, *aac(3)‐IId*, *aph(3′)‐Ia*, *aph(3″)‐Ib*, *aph(6)‐Id*, *fosA3*, *catA1*, *cmlA1*, *sul1*, *sul2*, *dfrA17*, *tet(B)*, *mdf(A)*	Q1, FIB, FII, N, X1	PQEU00000000
ECPET13	Striped Owl (*Asio clamator*)	212/‐	O18/O18ac:H49	*iss*, *lpfA*	AMC, CRO, CTX, CAZ, CPM, ATM, NAL	*bla*_CTX‐M‐55_, *bla*_TEM‐1B_, *mdf(A)*	FII, N	PQEV00000000
ECPET31	South American Coati (*Nasua nasua*)	58/155	O78:H21	*gad*, *iss*, *lpfA*	CRO, CTX, CAZ, CPM, ATM, CIP, GEN, KAN, TOB, STR, CLO, SUT, TET	*bla*_CTX‐M‐2_, *aadA1*, *aac(3)‐IV*, *aph(3″)‐Ib*, *aph(3′)‐Ia*, *aph(4)‐Ia*, *aph(6)‐Id*, *sul1*, *sul2*, *dfrA7*, *tet(A)*, *mdf(A)*	FIA, HI2, HI2A, Q1, FII	PQEW00000000
ECPET36	South American Coati (*Nasua nasua*)	1251/‐	O130:H26	*gad*	AMC, CRO, CTX, CAZ, CPM, ATM, CIP, NAL, GEN, KAN, TOB, STR, CLO, SUT	*bla*_CTX‐M‐15_, *bla*_TEM‐1B_, *qnrB1*, *aac(3)‐IIa*, *aac(6′)Ib‐cr*, *aadA1*, *aph(3″)‐Ib*, *aph(6)‐Id*, *catB3*, *catA1*, *sul2*, *dfrA1*, *dfrA14*, *mdf(A)*	HI2, HI2A, p0111	PQEX00000000
ICBUR6	Black Vulture (*Coragyps atratus*)	1158/31	O17/O44/O77:H34	*iss*, *astA*, *eilA*, *celb*, *iha*, *air*, *ireA*	CRO, CTX, CPM, ATM, KAN, TOB, CLO, SUL, TET	*bla*_CTX‐M‐2_, *qnrB19*, *aadA1*, *aph(3′)‐Ia*, *aph(6)‐Id*, *aph(3″`)‐Ib*, *catA1*, *sul1*, *sul2*, *tet(B)*, *mdf(A)*	FIB, FII, Col156	PPCS00000000
ICBUR15	Black Vulture (*Coragyps atratus*)	38/38	O86:H18	*gad*, *iss*, *astA*, *eilA*	CRO, CTX, CPM, ATM, CIP, NAL, TOB, STR, SUL	*bla*_CTX‐M‐14_, *aph(6)‐Id*, *aph(3″)‐Ib*, *sul2*, *mdf(A)*	l2	PPCU00000000
ICBUR20	Black Vulture (*Coragyps atratus*)	38/38	O86:H18	*iss*, *astA*, *eilA*, *air*	CRO, CTX, CPM, ATM, CIP, NAL, AMK, STR, SUL	*bla*_CTX‐M‐14_, *aph(6)‐Id*, *aph(3″)‐Ib*, *sul2*, *mdf(A)*	l2	PPCT00000000

Abbreviations: CC, clonal complex; ST, sequence type. Resistance phenotype: AMC, amoxicillin/clavulanic acid; AMK, amikacin; ATM, aztreonam; CAZ, ceftazidime; CIP, ciprofloxacin; CLO, chloramphenicol; CPM, cefepime; CRO, ceftriaxone; CTX, cefotaxime; GEN, gentamicin; KAN, kanamycin; NAL, nalidixic acid; STR, streptomycin; SUL, sulfonamide; SUT, trimethoprim‐sulfamethoxazole; TET, tetracycline; TOB, Tobramycin.

WGS analysis revealed six different serotypes (i.e. O18/O18ac:H49, O89/O162:H10, O78:H21, O130:H26, O17/O44/O77:H34, O86:H18). In this regard, the O86:H18 has been previously identified in diarrhoeagenic *E. coli* isolated from humans, in Brazil (Ghilardi, Gomes, & Trabulsi, 2001; Piva et al., 2003), and in Asian and African countries (Sonda et al., 2018; Suzuki et al., 2009). On the other hand, while *E. coli* O89/O162:H10 has been associated with hospital‐acquired infections, in Asian countries (Lin, Kuroda, Suzuki, & Mu, 2019; Nguyen et al., 2019), *E. coli* O18:H49 and O78:H21 have been reported in wild animals from Europe and Asia (Bai et al., 2013; Eggert et al., 2013). *Escherichia coli* O130:H26 and O17/O44/O77:H34 have been identified in human and animal samples from Asia, Europe, Australia, Antarctica and South America (Bettelheim et al., 2003; Delgado‐Blas, Ovejero, Abadia‐Patino, & Gonzalez‐Zorn, 2016; Ho, Tan, Ooi, Yeo, & Thong, 2013; Mora et al., 2018; Müller et al., 2007).

Virulome analysis revealed a diversity of virulence determinants, including *celb* (endonuclease colicin E2), *iha* (*irgA* homolog adhesin), *air* (enteroaggregative immunoglobulin repeat protein), *ireA* (siderophore receptor), *astA* (EAST1 toxin), *cma* (colicin M), *gad* (glutamate decarboxylase), *eilA* (*Salmonella HilA* homolog), *lpfA* (long polar fimbriae), *iroN* (enterobactin siderophore receptor protein) and *iss* (increased serum survival) (Table 1). Interestingly, *air*, *astA* and *eilA* genes have been found in enteroaggregative *E. coli* (EAEC) causing acute and chronic diarrhoea (Konno, Yatsuyanagi, & Saito, 2012; Nüesch‐Inderbinen, Hofer, Hachler, Beutin, & Stephan, 2013; Sheikh et al., 2006). The *lpfA* gene has been identified in enteropathogenic *E. coli* (EPEC), the most important diarrhoeal pathogen in paediatric patients (Afset et al., 2006).

In addition to *bla*_CTX‐M_‐type genes, resistome analysis confirmed that the *E. coli* strains carried other clinically relevant resistance genes to β‐lactams [*bla*_TEM‐1B_], aminoglycosides [*aadA1, aadA2, aadA5, aac(3)‐IId, aac(3)‐IIa*, *aac(3)‐IV, aac(6’)Ib‐cr, aph(3’)‐Ia, aph(3'')‐Ib, aph(3)‐Id, aph(4)‐Ia* and *aph(6)‐Id*], phenicols [*catA1, catB3* and *cmlA1*], tetracyclines [*tet(A)* and *tet(B*)], sulphonamides [*sul1* and *sul2*], trimethoprim [*dfrA1, dfrA7, dfrA14* and *dfrA17*], fosfomycin [*fosA3*], quinolones [*qnrB1, qnrB19* and *aac(6’)Ib‐cr*] and macrolides [*mdf(A)*]. Interestingly, in Brazil, there are only three studies regarding the plasmid‐mediated quinolone resistance *qnrB1* gene, which has been harboured by *K. pneumoniae* and *Enterobacter cloacae* (Scavuzzi et al., 2017; Viana et al., 2013), and *Enterobacter hormaechei* (Pereira et al., 2015). On the other hand, *qnrB19*, *bla*_CTX‐M‐2_, *bla*_CTX‐M‐15_ and *bla*_CTX‐M‐55_ genes have been carried by members of Enterobacterales genus isolated from human and non‐human hosts (Goldberg et al., 2019; Monte et al., 2019; Rocha, Pinto, & Barbosa, 2016; Sartori et al., 2017; Silva et al., 2018).

**FIGURE 1:**
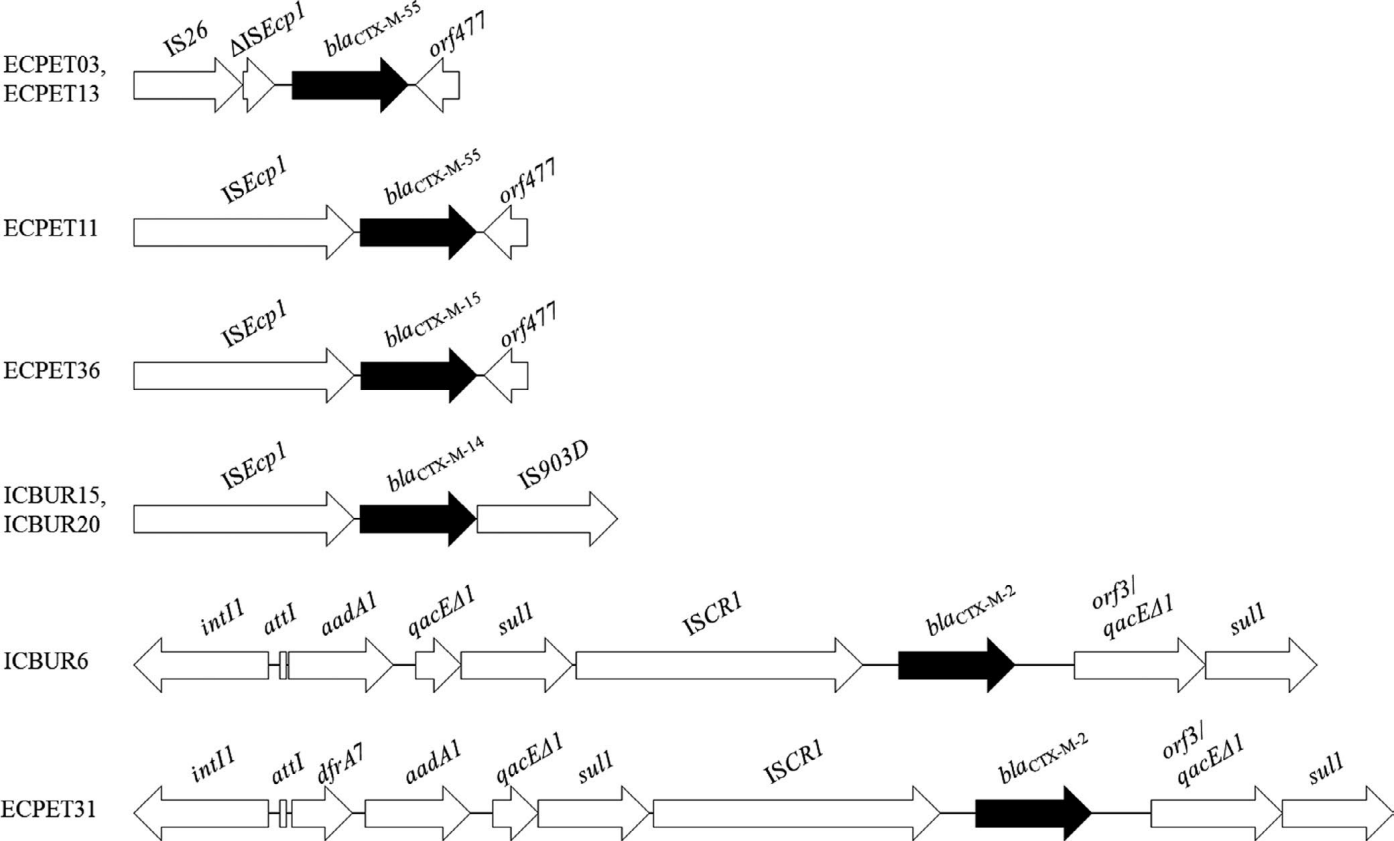
Schematic representation of the genetic context surrounding *bla*_CTX‐M_ genes in *Escherichia coli *strains from peri‐urban wild animals in Brazil. For ECPET03 and ECPET13 *E. coli* strains, the genetic array IS*26*‐ΔIS*Ecp1*‐*bla*_CTX‐M‐55_‐*orf477* was identified. In this regard, IS*26* belongs to IS*6* family, whereas *orf477* encodes a cupin fold metalloprotein of WbuC family. For ECPET11 and ECPET36 *E. coli* strains, carrying *bla*_CTX‐M‐55_ or *bla*_CTX‐M‐15_, respectively, IS*Ecp1* was upstream, whereas *orf477* was downstream of *bla*_CTX‐M_ genes. *E. coli* strains ICBUR15 and ICBUR20, and ECPET36 displayed international genetic arrays IS*Ecp1*‐*bla*_CTX‐M‐14_‐IS*903D* and IS*Ecp1*‐*bla*_CTX‐M‐15_‐*orf477*, respectively

Schematic representations of the genetic contexts surrounding *bla*_CTX‐M_‐type genes in *E. coli* strains are presented in Figure 1. International genetic contexts of *bla*_CTX‐M‐14_ (IS*Ecp1‐bla*_CTX‐M‐14_ *‐*IS*903D*) (Lartigue, Poirel, & Nordmann, 2004) and *bla*_CTX‐M‐15_ (IS*Ecp1‐bla*_CTX‐M‐15_ *‐orf477*) (Dhanji et al., 2011) were identified in *E. coli* strains ST38 (ICBUR15 and ICBUR20) and ST1251 (ECPET36) isolated from *C. atratus* and *N. nasua*, respectively. In addition, two different contexts were found surrounding the *bla*_CTX‐M‐55_. The typical structure IS*Ecp1‐bla*_CTX‐M‐55_ *‐orf477* (2,956 bp) was present in *E. coli* belonging to ST744 (ECPET11), whereas a similar array exhibiting a 243 bp with IS*Ecp1* truncated by an IS*26* upstream of the *bla*_CTX‐M‐55_ gene was found in *E. coli* strains ECPET3 and ECPET13 (ST212). Similar genetic contexts of *bla*_CTX‐M‐55_ have been reported in Enterobacteriaceae isolated from humans, animals and food animals (Hu et al., 2018; Lv et al., 2013). Furthermore, *bla*_CTX‐M‐2_ gene from *E. coli* strains ST58 (ECPET31) and ST1158 (ICBUR6) was present into complex class 1 integrons (9,456 and 8,879 bp, respectively), sharing 99.7% and 99.9% nucleotide identity with partial integrons of *E. coli* (GenBank: AM040710) (8,133 bp) and *K. pneumoniae* (GenBank: KY286109) (7,824 bp) isolated from French and Chilean hospitals, respectively.

Although different plasmid replicon types were found among CTX‐M‐producing *E. coli* strains, *bla*_CTX‐M_ genes were carried on IncF (FIA, FIB, and FII) plasmids, except *bla*_CTX‐M‐14_, which were carried on IncI2 plasmids. Most plasmids harbouring *bla*_CTX‐M_ genes were successfully transferred by conjugation (from *E. coli* donors ECPET3, ECPET13, ECPET36 and ICBUR6), or by transformation assays using plasmids from ICBUR15 and ICBUR20 strains. As previously reported, IncF plasmids have been widely associated with the spread of *bla*_CTX‐M‐15_, whereas IncF, IncK and IncI are commonly associated with *bla*_CTX‐M‐14_ and other *bla*_CTX‐M_‐type genes (Zhao & Hu, 2013). Regarding other plasmids identified in this study, IncN and IncHI2 have been related to the spread of *bla*_CTX‐M‐1_ and *bla*_CTX‐M‐9_, respectively, and IncQ1 or IncX plasmid has been responsible by dissemination of carbapenemase encoding genes (Cerdeira et al., 2019; Mollenkopf et al., 2017; Paul et al., 2017; Zhao & Hu, 2013).

**FIGURE 2:**
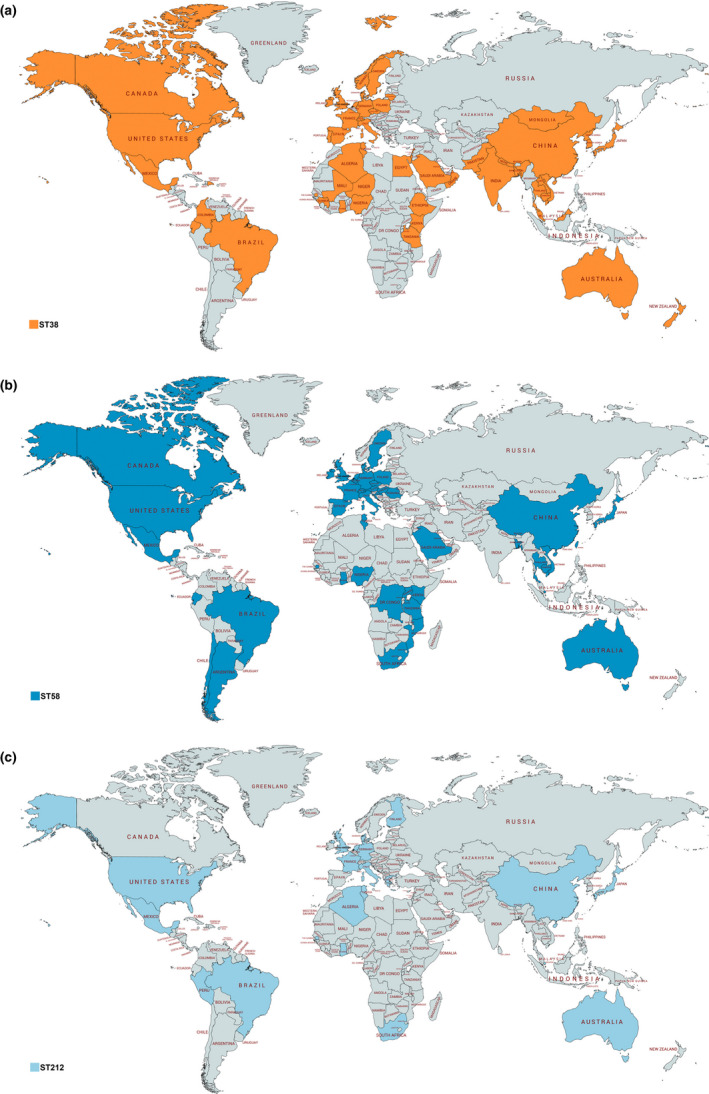
Global distribution of *Escherichia coli *belonging to sequence types (a) ST38, (b) ST58 and (c) ST212. This map was created using an online service (https://mapchart.net/) [Colour figure can be viewed at wileyonlinelibrary.com]

**FIGURE 3:**
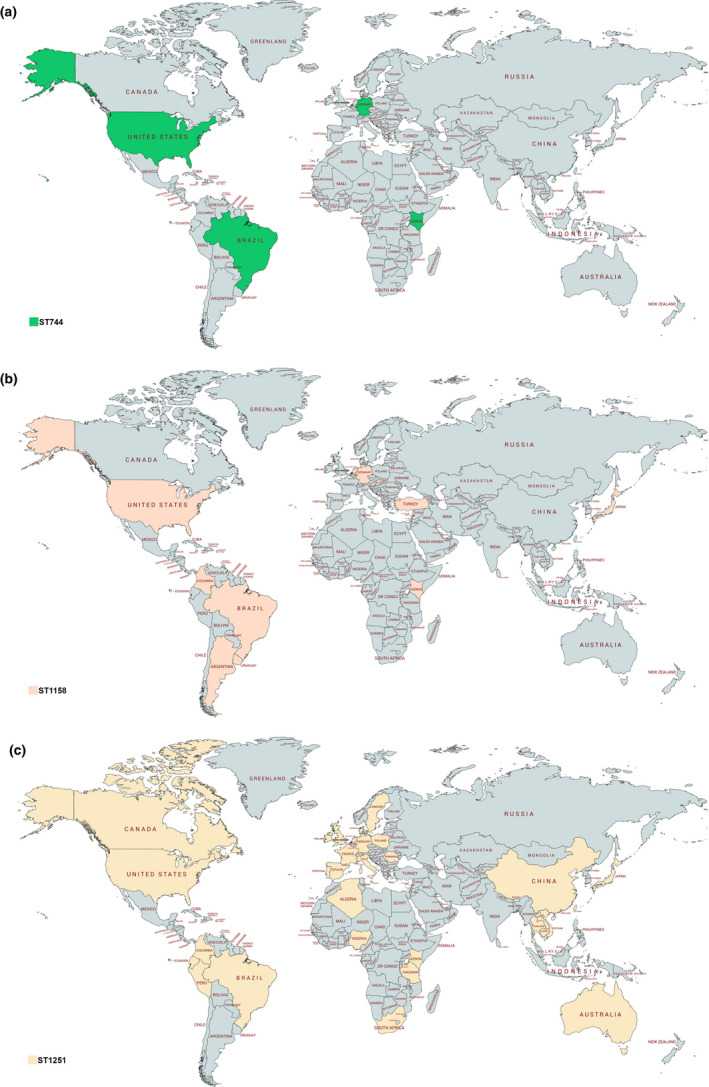
Global distribution of *Escherichia coli* belonging to sequence types (a) ST744, (b) ST1158 and (c) ST1251. This map was created using an online service (https://mapchart.net/) [Colour figure can be viewed at wileyonlinelibrary.com]

In this study, genomic analysis identified *E. coli* strains belonging to international ST38, ST58, ST212, ST744, ST1158 and ST1251 (Table 1). The global distribution of these *E. coli* clones is presented in Figures 2 and 3. The broadly distributed *E. coli* ST38 and ST744 have been reported in wildlife, farm animals and human samples from Europe, Africa, Asia, Australia and America, in general associated with the production of clinically significant beta‐lactamases (i.e. carbapenemases or ESBL) (Abraham et al., 2015; Belmahdi, Bakour, Al Bayssari, Touati, & Rolain, 2016; Guenther et al., 2017; Hasan et al., 2012; Ho et al., 2016; Mshana et al., 2011; Pitout, 2012; Poirel, Bernabeu, et al., 2011; Sellera et al., 2018; Stoesser et al., 2012; Yamamoto, Takano, Iwao, & Hishinuma, 2011). *Escherichia coli* ST38 has been frequently reported causing extraintestinal diseases, mainly bloodstream and urinary tract infections (Cao et al., 2014; Mendes, Jones, Woosley, Cattoir, & Castanheira, 2019; Pitout, 2012). In some cases, *E. coli* ST744 has been associated with plasmid‐mediated colistin resistance genes (*mcr‐1* and *mcr‐3*) (Haenni et al., 2018; Tacão et al., 2017). Furthermore, ESBL or CMY‐2‐producing *E. coli* ST212 and ST1158 were previously isolated from farm animals, animal production chain and humans (Cadona, Bustamante, Gonzalez, & Sanso, 2016; Castellanos et al., 2017; Maamar et al., 2016; Mo, Slettemeas, Berg, Norstrom, & Sunde, 2016; Steinsland, Lacher, Sommerfelt, & Whittam, 2010; Vignoli et al., 2016; Zurfluh et al., 2014). Carbapenemase or CMY‐2‐producing *E. coli* ST212 was also recovered from diseased companion animal and water environments (Tafoukt et al., 2017; Vingopoulou et al., 2014), whereas *E*. *coli* ST1158 carrying *bla*_CTX‐M_ was recovered from food animals (Vogt et al., 2014). Regarding *E. coli* ST1251, fluoroquinolone‐resistant strains have been reported in animal faeces and wastewater (Jamborova et al., 2015; Varela, Macedo, Nunes, & Manaia, 2015), as well as *mcr‐1*‐harbouring strains from food animals (Zurfluh et al., 2017). *Escherichia coli* belonging to ST58 has been globally reported from a variety of sources including food (Ben Said et al., 2015), polluted mangrove (Sacramento et al., 2018), poultry, hospital‐ and community‐acquired infections (Borges et al., 2019; McKinnon, Chowdhury, & Djordjevic, 2018) and bovine mastitis (Nüesch‐Inderbinen et al., 2019). Interestingly, ST58/CC155 frequently shares identical antimicrobial resistance patterns in both animal and human populations. Such evidence may significantly explain the successful establishment of this international lineage (Borges et al., 2019).

ESBL‐producing *E. coli* in wild animals begun to be documented in 2006, in Portugal (Costa et al., 2006), and then were rapidly observed in other countries from Europe, Africa, Asia, South America, North America and Australia (Allen et al., 2010; Wang et al., 2017). Predominantly, *E. coli*‐ and *K. pneumoniae*‐producing CTX‐M seem to be the most adapted to these hosts; however, the identification of ESBL genes in other different species of Enterobacterales has already been reported (Wang et al., 2017). In most of cases, animals became colonized in gastrointestinal tract without any evidences of infection, contributing for the silent dissemination of these critically important pathogens in natural environments.

A widely debated example is the occurrence of ESBL‐producing bacteria in migratory birds, which are probably involved in the spread of these pathogens through long distances, including natural reserves and pelagic areas with low anthropogenic impact (Ardiles‐Villegas et al., 2011; Cerdà‐Cuéllar et al., 2019). Otherwise, the role of peri‐urban wild animals as disseminators of bacterial pathogens has been so far neglected. In this study, all animals sampled lived in the transboundary area of São Paulo city, the most populated metropolitan region of Brazil, with about 21.5 million inhabitants, and one of the ten most populous metropolitan regions in the world. Even though the source of these bacterial isolates remains uncertain, wildlife is not directly exposed to antibiotics in most cases and other anthropogenic pathways of transmission, such as contact to contaminated water and predation of infected animals, should be considered (Wang et al., 2017). Yet, it is important to take in account that some highly polluted rivers cross this area, where KPC‐2‐ and ESBL‐producing *K. pneumoniae* isolates from water samples were previously reported (Cerdeira et al., 2017; Oliveira et al., 2014).

Since, in this study, ESBL‐positive isolates were recovered from predators with different ecological behaviours [i.e. vultures are scavengers and diurnal predators; owls are nocturnal hunters of small rodents; and coatis are remarkably well adapted predators, feeding on fruits, insects and small vertebrates], in order to investigate in more detail the genetic relatedness among these *E. coli* strains, core genome multilocus sequence typing (cgMLST) was performed by uploading the sequencing reads of the eight strains into cgMLSTFinder 1.1 (https://cge.cbs.dtu.dk/services/cgMLSTFinder/). Interestingly, two *E. coli* ST212 strains (ECPET3 and ECPET13) isolated from different hosts (black vulture and striped owl) were nested together (Figure S1). Remarkably, these strains also shared identical serotype, resistome and plasmidome. These findings suggest an adaptation of CTX‐M‐producing *E. coli* into the wildlife food chain and the versatility of these bacteria to colonize different hosts. Indeed, interspecific interactions among wild animals colonized by ESBL producers represent an incommensurable threat to ecosystem maintenance, since Enterobacteriaceae constitutes the gut microbiota of most endothermic animals (Madoshi et al., 2016). Thus, antimicrobial resistance must also be viewed as an ecological problem (Fuentes‐Castillo et al., 2019).

In conclusion, anthropogenic activities have been contributing for the dissemination of ESBL‐producing bacteria in wildlife. The occurrence of ESBL‐producing bacteria in peri‐urban wild animals from highly populated cities is a critical issue and deserves special attention. Therefore, continuous epidemiological and genomic surveillance studies are urgently required to determine routes of transmission of these bacteria in wildlife. Finally, while humans can negatively affect nature environments for contributing to the spread of MDR bacteria, animals could also disseminate these pathogens to humans in a continuous cycle.

## ETHICAL STATEMENT

The authors confirm that the ethical policies of the journal, as noted on the journal's author guidelines page, have been adhered to and the appropriate ethical review committee approval has been received.

## CONFLICT OF INTEREST

The authors have no conflict of interest to declare.

## Supporting information

Fig S1

## References

[tbed13558-bib-0001] Abraham, S. , Jordan, D. , Wong, H. S. , Johnson, J. R. , Toleman, M. A. , Wakeham, D. L. , … Trott, D. J. (2015). First detection of extended‐spectrum cephalosporin‐ and fluoroquinolone‐resistant *Escherichia coli* in Australian food‐producing animals. Journal of Global Antimicrobial Resistance, 3(4), 273–277. 10.1016/j.jgar.2015.08.002 27842872

[tbed13558-bib-0002] Afset, J. E. , Bruant, G. , Brousseau, R. , Harel, J. , Anderssen, E. , Bevanger, L. , & Bergh, K. (2006). Identification of virulence genes linked with diarrhea due to atypical enteropathogenic *Escherichia coli* by DNA microarray analysis and PCR. Journal of Clinical Microbiology, 44(10), 3703–3711. 10.1128/JCM.00429-06 17021100 PMC1594803

[tbed13558-bib-0003] Allen, K. H. , Donato, J. , Wang, H. H. , Cloud‐Hansen, K. A. , Davies, J. , & Handelsman, J. (2010). Call of the wild: Antibiotic resistance genes in natural environments. Nature Reviews Microbiology, 8(4), 251–259. 10.1038/nrmicro2312 20190823

[tbed13558-bib-0004] Ardiles‐Villegas, K. , González‐Acuña, D. , Waldenström, J. , Olsen, B. , & Hernández, J. (2011). Antibiotic resistance patterns in fecal bacteria isolated from Christmas shearwater (*Puffinus nativitatis*) and masked booby (*Sula dactylatra*) at remote Easter Island. Avian Diseases, 55(3), 486–489. 10.1637/9619-122010-ResNote.1 22017052

[tbed13558-bib-0005] Bai, X. , Zhao, A. , Lan, R. , Xin, Y. , Xie, H. , Meng, Q. , … Xiong, Y. (2013). Shiga toxin‐producing *Escherichia coli* in yaks (*Bos grunniens*) from the Qinghai‐Tibetan Plateau, China. PLoS ONE, 8(6), e65537 10.1371/journal.pone.0065537 23776496 PMC3679134

[tbed13558-bib-0006] Bankevich, A. , Nurk, S. , Antipov, D. , Gurevich, A. A. , Dvorkin, M. , Kulikov, A. S. , … Pevzner, P. A. (2012). SPAdes: A new genome assembly algorithm and its applications to single‐cell sequencing. Journal of Computational Biology, 19(5), 455–477. 10.1089/cmb.2012.0021 22506599 PMC3342519

[tbed13558-bib-0007] Belmahdi, M. , Bakour, S. , Al Bayssari, C. , Touati, A. , & Rolain, J. M. (2016). Molecular characterisation of extended‐spectrum β‐lactamase‐ and plasmid AmpC‐producing *Escherichia coli* strains isolated from broilers in Béjaïa, Algeria. Journal of Global Antimicrobial Resistance, 6, 108–112. 10.1016/j.jgar.2016.04.006 27530851

[tbed13558-bib-0008] Ben Said, L. , Jouini, A. , Klibi, N. , Dziri, R. , Alonso, C. A. , Boudabous, A. , … Torres, C. (2015). Detection of extended‐spectrum beta‐lactamase (ESBL)‐producing Enterobacteriaceae in vegetables, soil and water of the farm environment in Tunisia. International Journal of Food Microbiology, 16(203), 86–92. 10.1016/j.ijfoodmicro.2015.02.023 25791254

[tbed13558-bib-0009] Bettelheim, K. A. , Beutin, L. , Gleier, K. , Pearce, J. L. , Luke, R. K. , & Zimmermann, S. (2003). Serotypes of *Escherichia coli* isolated from healthy infants in Berlin, Germany and Melbourne, Australia. Comparative Immunology, Microbiology & Infectious Diseases, 26(1), 55–63. 10.1016/s0147-9571(02)00015-2 12602687

[tbed13558-bib-0010] Borges, C. A. , Tarlton, N. J. , & Riley, L. W. (2019). Escherichia coli from commercial broiler and backyard chickens share sequence types, antimicrobial resistance profiles, and resistance genes with human extraintestinal pathogenic *Escherichia coli* . Foodborne Pathogens and Disease, 16(12), 813–822. 10.1089/fpd.2019.2680 31411497

[tbed13558-bib-0011] Brolund, A. (2014). Overview of ESBL‐producing enterobacteriaceae from nordic perspective. Infection Ecology and Epidemology, 4(1), 24555 10.3402/iee.v4.24555 PMC4185132 25317262

[tbed13558-bib-0012] Cadona, J. S. , Bustamante, A. V. , Gonzalez, J. , & Sanso, A. M. (2016). Genetic relatedness and novel sequence types of non‐O157 shiga toxin‐producing *Escherichia coli* strains isolated in Argentina. Frontiers in Cellular and Infection Microbiology, 6, 93 10.3389/fcimb.2016.00093 27625995 PMC5003923

[tbed13558-bib-0013] Cao, X. , Zhang, Z. , Shen, H. , Ning, M. , Chen, J. , Wei, H. , & Zhang, K. (2014). Genotypic characteristics of multidrug‐resistant Escherichia coli isolates associated with urinary tract infections. Acta Pathologica, Microbiologica, Et Immunologica Scandinavica, 122(11), 1088–1095. 10.1111/apm.12260 24698634

[tbed13558-bib-0014] Castellanos, L. R. , Donado‐Godoy, P. , León, M. , Clavijo, V. , Arevalo, A. , Bernal, J. F. , … Hordijk, J. (2017). High heterogeneity of *Escherichia coli* sequence types harbouring ESBL/AmpC Genes on IncI1 plasmids in the colombian poultry chain. PLoS ONE, 12(1), e0170777 10.1371/journal.pone.0170777 28125687 PMC5268450

[tbed13558-bib-0015] Cerdà‐Cuéllar, M. , Moré, E. , Ayats, T. , Aguilera, M. , Muñoz‐González, S. , Antilles, N. , … González‐Solís, J. (2019). Do humans spread zoonotic enteric bacteria in Antarctica? Science of the Total Environment, 654, 190–196. 10.1016/j.scitotenv.2018.10.272 30445320

[tbed13558-bib-0016] Cerdeira, L. , Fernandes, M. R. , Ienne, S. , Souza, T. A. , de O. Garcia, D. , & Lincopan, N. (2017). Draft genome sequence of an environmental multidrug‐resistant *Klebsiella pneumoniae* ST340/CC258 harbouring blaCTX‐M‐15 and blaKPC‐2 genes. Journal of Global Antimicrobial Resistance, 8, 108–109. 10.1016/j.jgar.2016.12.001 28082142

[tbed13558-bib-0017] Cerdeira, L. T. , Lam, M. M. C. , Wyres, K. L. , Wick, R. R. , Judd, L. M. , Lopes, R. , … Lincopan, N. (2019). Small IncQ1 and Col‐Like plasmids harboring blaKPC‐2 and non‐Tn4401 elements (NTEKPC‐IId) in high‐risk lineages of *Klebsiella pneumoniae* CG258. Antimicrobial Agents and Chemotherapy, 63(3), e02140–e2218. 10.1128/AAC.02140-18 30602517 PMC6395902

[tbed13558-bib-0018] Clinical and Laboratory Standards Institute . (2017). Performance standards for antimicrobial susceptibility testing; twenty‐seven informational supplement. CLSI document M100–S27. Wayne, PA: CLSI.

[tbed13558-bib-0019] Clinical Laboratory Standards Institute . (2015). Performance standards for antimicrobial disk and dilution susceptibility tests for bacteria isolated from animals (3rd edition). CLSI Supplement VET01S. Wayne, PA: CLSI.

[tbed13558-bib-0020] Costa, D. , Poeta, P. , Sáenz, Y. , Vinué, L. , Rojo‐Bezares, B. , Jouini, A. , … Torres, C. (2006). Detection of Escherichia coli harbouring extended‐spectrum beta‐lactamases of the CTX‐M, TEM and SHV classes in faecal samples of wild animals in Portugal. Journal of Antimicrobial Chemotherapy, 58(6), 1311–1312. 10.1093/jac/dkl415 17023496

[tbed13558-bib-0021] Delgado‐Blas, J. F. , Ovejero, C. M. , Abadia‐Patino, L. , & Gonzalez‐Zorn, B. (2016). Coexistence of mcr‐1 and blaNDM‐1 in Escherichia coli from Venezuela. Antimicrobial Agents and Chemotherapy, 60(10), 6356–6358. 10.1128/AAC.01319-16 27431212 PMC5038285

[tbed13558-bib-0022] Dhanji, H. , Patel, R. , Wall, R. , Doumith, M. , Patel, B. , Hope, R. , … Woodford, N. (2011). Variation in the genetic environments of blaCTX‐M‐15 in Escherichia coli from the faeces of travellers returning to the United Kingdom. Journal of Antimicrobial Chemotherapy, 66(5), 1005–1012. 10.1093/jac/dkr041 21393166

[tbed13558-bib-0023] Dropa, M. , Lincopan, N. , Balsalobre, L. C. , Oliveira, D. E. , Moura, R. A. , Fernandes, M. R. , … Matté, M. H. (2016). Genetic background of novel sequence types of CTX‐M‐8‐ and CTX‐M‐15‐producing Escherichia coli and Klebsiella pneumoniae from public wastewater treatment plants in São Paulo, Brazil. Environmental Science and Pollution Research, 23(5), 4953–4958. 10.1007/s11356-016-6079-5 26782324

[tbed13558-bib-0024] Eggert, M. , Stuber, E. , Heurich, M. , Fredriksson‐Ahomaa, M. , Burgos, Y. , Beutin, L. , & Martlbauer, E. (2013). Detection and characterization of Shiga toxin‐producing *Escherichia coli* in faeces and lymphatic tissue of free‐ranging deer. Epidemiology & Infection, 141(2), 251–259. 10.1017/S0950268812000246 22370185 PMC9152051

[tbed13558-bib-0025] Fernandes, M. R. , Sellera, F. P. , Moura, Q. , Gaspar, V. C. , Cerdeira, L. , & Lincopan, N. (2018). International high‐risk clonal lineages of CTX‐M‐producing *Escherichia coli* F‐ST648 in free‐roaming cats, South America. Infection, Genetics and Evolution, 66, 48–51. 10.1016/j.meegid.2018.09.009 30227226

[tbed13558-bib-0026] Fuentes‐Castillo, D. , Farfán‐López, M. , Esposito, F. , Moura, Q. , Fernandes, M. R. , Lopes, R. , … Lincopan, N. (2019). Wild owls colonized by international clones of extended‐spectrum β‐lactamase (CTX‐M)‐producing *Escherichia coli* and Salmonella Infantis in the Southern Cone of America. Science of the Total Environment, 674, 554–562. 10.1016/j.scitotenv.2019.04.149 31022545

[tbed13558-bib-0027] Ghilardi, A. C. , Gomes, T. A. , & Trabulsi, L. R. (2001). Production of cytolethal distending toxin and other virulence characteristics of *Escherichia coli* strains of serogroup O86. Memórias do Instituto Oswaldo Cruz, 96(5), 703–708. 10.1590/S0074-02762001000500022 11500775

[tbed13558-bib-0028] Goldberg, D. W. , Fernandes, M. R. , Sellera, F. P. , Costa, D. G. C. , Loureiro Bracarense, A. P. , & Lincopan, N. (2019). Genetic background of CTX‐M‐15‐producing *Enterobacter hormaechei* ST114 and *Citrobacter freundii* ST265 co‐infecting a free‐living green turtle (*Chelonia mydas*). Zoonoses Public Health, 66(5), 540–545. 10.1111/zph.12572 30843359

[tbed13558-bib-0029] Guenther, S. , Semmler, T. , Stubbe, A. , Stubbe, M. , Wieler, L. H. , & Schaufler, K. (2017). Chromosomally encoded ESBL genes in *Escherichia coli* of ST38 from Mongolian wild birds. Journal of Antimicrobial Chemotherapy, 72(5), 1310–1313. 10.1093/jac/dkx006 28158613

[tbed13558-bib-0030] Haenni, M. , Beyrouthy, R. , Lupo, A. , Chatre, P. , Madec, J. Y. , & Bonnet, R. (2018). Epidemic spread of *Escherichia coli* ST744 isolates carrying mcr‐3 and blaCTX‐M‐55 in cattle in France. Journal of Antimicrobial Chemotherapy., 73(2), 533–536. 10.1093/jac/dkx418 29182716 PMC5890774

[tbed13558-bib-0031] Hasan, B. , Sandegren, L. , Melhus, A. , Drobni, M. , Hernandez, J. , Waldenstrom, J. , … Olsen, B. (2012). Antimicrobial drug‐resistant *Escherichia coli* in wild birds and free‐range poultry, Bangladesh. Emerging Infectious Diseases, 18(12), 2055–2058. 10.3201/eid1812.120513 23171693 PMC3557866

[tbed13558-bib-0032] Ho, P. L. , Cheung, Y. Y. , Wang, Y. , Lo, W. U. , Lai, E. L. , Chow, K. H. , & Cheng, V. C. (2016). Characterization of carbapenem‐resistant *Escherichia coli* and *Klebsiella pneumoniae* from a healthcare region in Hong Kong. European Journal of Clinical Microbiology & Infectious Diseases, 35(3), 379–385. 10.1007/s10096-015-2550-3 26740321

[tbed13558-bib-0033] Ho, W. S. , Tan, L. K. , Ooi, P. T. , Yeo, C. C. , & Thong, K. L. (2013). Prevalence and characterization of verotoxigenic‐*Escherichia coli* isolates from pigs in Malaysia. BMC Veterinary Research, 9, 109 10.1186/1746-6148-9-109 23731465 PMC3681573

[tbed13558-bib-0034] Hu, X. , Gou, J. , Guo, X. , Cao, Z. , Li, Y. , Jiao, H. , … Tian, F. (2018). Genetic contexts related to the diffusion of plasmid‐mediated CTX‐M‐55 extended‐spectrum beta‐lactamase isolated from Enterobacteriaceae in China. Annals of Clinical Microbiology and Antimicrobials, 17(1), 12 10.1186/s12941-018-0265-x 29571292 PMC5865355

[tbed13558-bib-0035] Inoue, H. , Nojima, H. , & Okayama, H. (1990). High efficiency transformation of *Escherichia coli* with plasmids. Gene, 96(1), 23–28. 10.1016/0378-1119(90)90336-p 2265755

[tbed13558-bib-0036] Jamborova, I. , Dolejska, M. , Vojtech, J. , Guenther, S. , Uricariu, R. , Drozdowska, J. , … Literak, I. (2015). Plasmid‐mediated resistance to cephalosporins and fluoroquinolones in various *Escherichia coli* sequence types isolated from rooks wintering in Europe. Applied Environmental Microbiology, 81(2), 648–657. 10.1128/AEM.02459-14 25381245 PMC4277596

[tbed13558-bib-0037] Konno, T. , Yatsuyanagi, J. , & Saito, S. (2012). Virulence gene profiling of enteroaggregative *Escherichia coli* heat‐stable enterotoxin 1‐harboring *E. coli* (EAST1EC) derived from sporadic diarrheal patients. FEMS Immunology and Medical Microbiology, 64(3), 314–320. 10.1111/j.1574-695X.2011.00913.x 22106844

[tbed13558-bib-0038] Lartigue, M. F. , Poirel, L. , & Nordmann, P. (2004). Diversity of genetic environment of blaCTX‐M genes. FEMS Microbiology Letters, 234(2), 201–207. 10.1016/j.femsle.2004.01.051 15135523

[tbed13558-bib-0039] Lin, Y. C. , Kuroda, M. , Suzuki, S. , & Mu, J. J. (2019). Emergence of an *Escherichia coli* strain co‐harbouring mcr‐1 and blaNDM‐9 from a urinary tract infection in Taiwan. Journal of Global Antimicrobial Resistance, 16, 286–290. 10.1016/j.jgar.2018.10.003 30312830

[tbed13558-bib-0040] Liu, Y.‐Y. , Wang, Y. , Walsh, T. R. , Yi, L.‐X. , Zhang, R. , Spencer, J. , … Shen, J. (2016). Emergence of plasmid‐mediated colistin resistance mechanism MCR‐1 in animals and human beings in China: A microbiological and molecular biological study. The Lancet Infectious Diseases, 16(2), 161–168. 10.1016/S1473-3099(15)00424-7 26603172

[tbed13558-bib-0041] Lv, L. , Partridge, S. R. , He, L. , Zeng, Z. , He, D. , Ye, J. , & Liu, J. H. (2013). Genetic characterization of IncI2 plasmids carrying blaCTX‐M‐55 spreading in both pets and food animals in China. Antimicrobial Agents and Chemotherapy, 57(6), 2824–2827. 10.1128/AAC.02155-12 23478963 PMC3716176

[tbed13558-bib-0042] Maamar, E. , Hammami, S. , Alonso, C. A. , Dakhli, N. , Abbassi, M. S. , Ferjani, S. , … Boutiba‐Ben Boubaker, I. (2016). High prevalence of extended‐spectrum and plasmidic ampc beta‐lactamase‐producing *Escherichia coli* from poultry in Tunisia. International Journal of Food Microbiology, 231, 69–75. 10.1016/j.ijfoodmicro 27220012

[tbed13558-bib-0043] Madoshi, B. P. , Kudirkiene, E. , Mtambo, M. M. , Muhairwa, A. P. , Lupindu, A. M. , & Olsen, J. E. (2016). Characterisation of commensal *Escherichia coli* isolated from apparently healthy cattle and their attendants in Tanzania. PLoS ONE, 11(12), e0168160 10.1371/journal.pone.0168160 27977751 PMC5158034

[tbed13558-bib-0044] Magiorakos, A.‐P. , Srinivasan, A. , Carey, R. B. , Carmeli, Y. , Falagas, M. E. , Giske, C. G. , … Monnet, D. L. (2012). Multidrug‐resistant, extensively drug‐resistant and pandrug‐resistant bacteria: An international expert proposal for interim standard definitions for acquired resistance. Clinical Microbiology and Infection, 18(3), 268–281. 10.1111/j.1469-0691.2011.03570.x 21793988

[tbed13558-bib-0045] McKinnon, J. , Roy Chowdhury, P. , & Djordjevic, S. P. (2018). Genomic analysis of multidrug‐resistant *Escherichia coli* ST58 causing urosepsis. International Journal of Antimicrobial Agents, 52(3), 430–435. 10.1016/j.ijantimicag.2018.06.017 29966679

[tbed13558-bib-0046] Mendes, R. E. , Jones, R. N. , Woosley, L. N. , Cattoir, V. , & Castanheira, M. (2019). Application of next‐generation sequencing for characterization of surveillance and clinical trial isolates: Analysis of the distribution of β‐lactamase resistance genes and lineage background in the United States. Open Forum Infectious Diseases, 6(1), S69–S78. 10.1093/ofid/ofz004 30895217 PMC6419912

[tbed13558-bib-0047] Minarini, L. A. , Poirel, L. , Trevisani, N. A. , Darini, A. L. , & Nordmann, P. (2009). Predominance of CTX‐M‐type extended‐spectrum beta‐lactamase genes among enterobacterial isolates from outpatients in Brazil. Diagnostic Microbiology and Infectious Disease, 65(2), 202–206. 10.1016/j.diagmicrobio.2009.05.021 19748435

[tbed13558-bib-0048] Mo, S. S. , Slettemeas, J. S. , Berg, E. S. , Norstrom, M. , & Sunde, M. (2016). Plasmid and host strain characteristics of *Escherichia coli* resistant to extended‐spectrum cephalosporins in the Norwegian broiler production. PLoS ONE, 11(4), e015401911 10.1371/journal.pone.0154019 PMC4844124 27111852

[tbed13558-bib-0049] Mollenkopf, D. F. , Stull, J. W. , Mathys, D. A. , Bowman, A. S. , Feicht, S. M. , Grooters, S. V. , … Wittum, T. E. (2017). Carbapenemase‐producing Enterobacteriaceae recovered from the environment of a swine farrow‐to‐finish operation in the United States. Antimicrobial Agents and Chemotherapy, 61(2), e01298–e1316. 10.1128/AAC.01298-16 PMC5278694 27919894

[tbed13558-bib-0050] Monte, D. F. , Lincopan, N. , Berman, H. , Cerdeira, L. , Keelara, S. , Thakur, S. , … Landgraf, M. (2019). Genomic features of high‐priority *Salmonella enterica* serovars circulating in the food production chain, Brazil, 2000–2016. Scientific Reports, 9(1), 11058 10.1038/s41598-019-45838-0 31363103 PMC6667439

[tbed13558-bib-0051] Mora, A. , García‐Peña, F. J. , Alonso, M. P. , Pedraza‐Diaz, S. , Ortega‐Mora, L. M. , Garcia‐Parraga, D. , … Blanco, J. (2018). Impact of human‐associated *Escherichia coli* clonal groups in Antarctic pinnipeds: Presence of ST73, ST95, ST141 and ST131. Scientific Reports, 8(1), 4678 10.1038/s41598-018-22943-0 29549276 PMC5856829

[tbed13558-bib-0052] Mshana, S. E. , Imirzalioglu, C. , Hain, T. , Domann, E. , Lyamuya, E. F. , & Chakraborty, T. (2011). Multiple st clonal complexes, with a predominance of ST131, of *Escherichia coli* harbouring blaCTX‐M‐15 in a tertiary hospital in Tanzania. Clinical Microbiology and Infection, 17(8), 1279–1282. 10.1111/j.1469-0691.2011.03518.x 21595794

[tbed13558-bib-0053] Müller, D. , Greune, L. , Heusipp, G. , Karch, H. , Fruth, A. , Tschape, H. , & Schmidt, M. A. (2007). Identification of unconventional intestinal pathogenic *Escherichia coli* isolates expressing intermediate virulence factor profiles by using a novel single‐step multiplex PCR. Applied and Environmental Microbiology, 73(10), 3380–3390. 10.1128/AEM.02855-06 17400780 PMC1907121

[tbed13558-bib-0054] Muzaheed, , Doi, Y. , Adams‐Haduch, J. M. , Endimiani, A. , Sidjabat, H. E. , Gaddad, S. M. , & Paterson, D. L. (2008). High prevalence of CTX‐M‐15‐producing *Klebsiella pneumoniae* among inpatients and outpatients with urinary tract infection in Southern India. Journal of Antimicrobial Chemotherapy, 61(6), 1393–1394. 10.1093/jac/dkn109 18356153 PMC2736628

[tbed13558-bib-0055] Nguyen, L. P. , Pinto, N. A. , Vu, T. N. , Mai, H. , Pham, A. H. T. , Lee, H. , … Yong, D. (2019). Resistome profiles, plasmid typing, and whole‐genome phylogenetic tree analyses of blaNDM‐9 and mcr‐1 co‐harboring *Escherichia coli* ST617 from a patient without a history of farm exposure in Korea. Pathogens, 8(4), E212 10.3390/pathogens8040212 31683614 PMC6963575

[tbed13558-bib-0056] Nuesch‐Inderbinen, M. T. , Hofer, E. , Hachler, H. , Beutin, L. , & Stephan, R. (2013). Characteristics of enteroaggregative *Escherichia coli* isolated from healthy carriers and from patients with diarrhoea. Journal of Medical Microbiology, 62(12), 1828–1834. 10.1099/jmm.0.065177-0 24008499

[tbed13558-bib-0057] Nüesch‐Inderbinen, M. , Käppeli, N. , Morach, M. , Eicher, C. , Corti, S. , & Stephan, R. (2019). Molecular types, virulence profiles and antimicrobial resistance of *Escherichia coli* causing bovine mastitis. Veterinary Record Open, 6(1), e000369 10.1136/vetreco-2019-000369 31897302 PMC6924703

[tbed13558-bib-0058] Oliveira, S. , Moura, R. A. , Silva, K. C. , Pavez, M. , McCulloch, J. A. , Dropa, M. , … Lincopan, N. (2014). Isolation of KPC‐2‐producing *Klebsiella pneumoniae* strains belonging to the high‐risk multiresistant clonal complex 11 (ST437 and ST340) in urban rivers. Journal of Antimicrobial Chemotherapy, 69(3), 849–852. 10.1093/jac/dkt431 24159156

[tbed13558-bib-0059] Pardon, B. , Smet, A. , Butaye, P. , Argudín, M. A. , Valgaeren, B. , Catry, B. , … Deprez, P. (2015). Nosocomial intravascular catheter infections with extended‐spectrum beta‐lactamase‐producing *Escherichia coli* in calves after strain introduction from a commercial herd. Transboundary and Emerging Diseases, 64(1), 130–136. 10.1111/tbed.12352.64:130-136 25903854 PMC7169822

[tbed13558-bib-0060] Paul, D. , Bhattacharjee, A. , Ingti, B. , Choudhury, N. A. , Maurya, A. P. , Dhar, D. , & Chakravarty, A. (2017). Occurrence of blaNDM‐7 within IncX3‐type plasmid of *Escherichia coli* from India. Journal of Infection and Chemotherapy, 23(4), 206–210. 10.1016/j.jiac.2016.12.009 28131738

[tbed13558-bib-0061] Pereira, P. S. , Borghi, M. , Albano, R. M. , Lopes, J. C. , Silveira, M. C. , Marques, E. A. , … Carvalho‐Assef, A. P. (2015). Coproduction of NDM‐1 and KPC‐2 in *Enterobacter hormaechei* from Brazil. Microbiology Drug Resistance, 21(2), 234–236. 10.1089/mdr.2014.0171 25473727

[tbed13558-bib-0062] Pitout, J. D. (2012). Extraintestinal pathogenic *Escherichia coli*: A combination of virulence with antibiotic resistance. Frontiers in Microbiology, 3, 9 10.3389/fmicb.2012.00009 22294983 PMC3261549

[tbed13558-bib-0063] Piva, I. C. , Pereira, A. L. , Ferraz, L. R. , Silva, R. S. N. , Vieira, A. C. , Blanco, J. E. , … Giugliano, L. G. (2003). Virulence markers of enteroaggregative *Escherichia coli* isolated from children and adults with diarrhea in Brasilia, Brazil. Journal of Clinical Microbiology, 41(5), 1827–1832. 10.1128/jcm.41.5.1827-1832.2003 12734212 PMC154701

[tbed13558-bib-0064] Poirel, L. , Bernabeu, S. , Fortineau, N. , Podglajen, I. , Lawrence, C. , & Nordmann, P. (2011). Emergence of OXA‐48‐producing *Escherichia coli* clone ST38 in France. Antimicrobial Agents and Chemotherapy, 55(10), 4937–4938. 10.1128/AAC.00413-11 21768512 PMC3186974

[tbed13558-bib-0065] Poirel, L. , Walsh, T. R. , Cuvillier, V. , & Nordmann, P. (2011). Multiplex PCR for detection of acquired carbapenemase genes. Diagnostic Microbiology and Infectious Disease, 70(1), 119–123. 10.1016/j.diagmicrobio.2010.12.002 21398074

[tbed13558-bib-0066] Radhouani, H. , Silva, N. , Poeta, P. , Torres, C. , Correia, S. , & Igrejas, G. (2014). Potential impact of antimicrobial resistance in wildlife, environment, and human health. Frontiers in Microbiology, 23, 1–12. 10.3389/fmicb.2014.00023 PMC3913889 24550896

[tbed13558-bib-0067] Rocha, F. R. , Pinto, V. P. , & Barbosa, F. C. (2016). The Spread of CTX‐M‐type extended‐spectrum β‐lactamases in Brazil: A systematic review. Microbial Drug Resistance, 22(4), 301–311. 10.1089/mdr.2015.0180 26669767

[tbed13558-bib-0068] Sacramento, A. G. , Fernandes, M. R. , Sellera, F. P. , Muñoz, M. E. , Vivas, R. , Dolabella, S. S. , & Lincopan, N. (2018). Genomic analysis of MCR‐1 and CTX‐M‐8 co‐producing *Escherichia coli* ST58 isolated from a polluted mangrove ecosystem in Brazil. Journal of Global Antimicrobial Resistance, 15, 288–289. 10.1016/j.jgar.2018.10.024 30404044

[tbed13558-bib-0069] Saladin, M. , Cao, V. T. , Lambert, T. , Donay, J. L. , Herrmann, J. L. , Ould‐Hocine, Z. , … Arlet, G. (2002). Diversity of CTX‐M beta‐lactamases and their promoter regions from Enterobacteriaceae isolated in three Parisian hospitals. FEMS Microbiology Letters, 209(2), 161–168. 10.1111/j.1574-6968.2002.tb11126.x 12007800

[tbed13558-bib-0070] Sambrook, J. , & Russel, D. W. (2001). Molecular cloning: A laboratory manual (Vol. 3, 3rd ed., p. 2100). New York: Cold Spring Harbor Laboratory Press.

[tbed13558-bib-0071] Sartori, L. , Fernandes, M. R. , Ienne, S. , de Souza, T. A. , Gregory, L. , Cerdeira, L. , & Lincopan, N. (2017). Draft genome sequences of two fluoroquinolone‐resistant CTX‐M‐15‐producing *Escherichia coli* ST90 (ST23 complex) isolated from a calf and a dairy cow in South America. Journal of Global Antimicrobial Resistance, 11, 145–147. 10.1016/j.jgar.2017.10.009 29111480

[tbed13558-bib-0072] Scavuzzi, A. M. L. , Maciel, M. A. V. , de Melo, H. R. L. , Alves, L. C. , Brayner, F. A. , & Lopes, A. C. S. (2017). Occurrence of qnrB1 and qnrB12 genes, mutation in gyrA and ramR, and expression of efflux pumps in isolates of *Klebsiella pneumoniae* carriers of blaKPC‐2. Journal of Medical Microbiology, 66(4), 477–484. 10.1099/jmm.0.000452 28425875

[tbed13558-bib-0073] Sellera, F. P. (2019). Epidemiological implications of drug‐resistant bacteria in wildlife rehabilitation centers. Journal of Infection and Public Health, 12(5), 748–749. 10.1016/j.jiph.2019.06.002 31230952

[tbed13558-bib-0074] Sellera, F. P. , Fernandes, M. R. , Moura, Q. , Carvalho, M. P. N. , & Lincopan, N. (2018). Extended‐spectrum‐β‐lactamase (CTX‐M)‐producing *Escherichia coli* in wild fishes from a polluted area in the Atlantic Coast of South America. Marine Pollution Bulletin, 135, 183–186. 10.1016/j.marpolbul.2018.07.012 30301029

[tbed13558-bib-0075] Sheikh, J. , Dudley, E. G. , Sui, B. , Tamboura, B. , Suleman, A. , & Nataro, J. P. (2006). EilA, a HilA‐like regulator in enteroaggregative *Escherichia coli* . Molecular Microbiology, 61(2), 338–350. 10.1111/j.1365-2958.2006.05234.x 16762026

[tbed13558-bib-0076] Siguier, P. , Perochon, J. , Lestrade, L. , Mahillon, J. , & Chandler, M. (2006). ISfinder: The reference centre for bacterial insertion sequences. Nucleic Acids Research, 34, D32–36. 10.1093/nar/gkj014 16381877 PMC1347377

[tbed13558-bib-0077] Silva, M. M. , Fernandes, M. R. , Sellera, F. P. , Cerdeira, L. , Medeiros, L. K. G. , Garino, F. , … Lincopan, N. (2018). Multidrug‐resistant CTX‐M‐15‐producing *Klebsiella pneumoniae* ST231 associated with infection and persistent colonization of dog. Diagnostic Microbiology and Infectious Disease, 92(3), 259–261. 10.1016/j.diagmicrobio.2018.06.012 30025966

[tbed13558-bib-0078] Sonda, T. , Kumburu, H. , van Zwetselaar, M. , Alifrangis, M. , Mmbaga, B. T. , Aarestrup, F. M. , … Lund, O. (2018). Whole genome sequencing reveals high clonal diversity of *Escherichia coli* isolated from patients in a tertiary care hospital in Moshi, Tanzania. Antimicrobial Resistance & Infection Control, 7, 72 10.1186/s13756-018-0361-x 29977533 PMC5992844

[tbed13558-bib-0079] Steinsland, H. , Lacher, D. W. , Sommerfelt, H. , & Whittam, T. S. (2010). Ancestral lineages of human enterotoxigenic *Escherichia coli* . Journal of Clinical Microbiology, 48(8), 2916–2924. 10.1128/JCM.02432-09 20534806 PMC2916599

[tbed13558-bib-0080] Stoesser, N. , Crook, D. W. , Moore, C. E. , Phetsouvanh, R. , Chansamouth, V. , Newton, P. N. , & Jones, N. (2012). Characteristics of CTX‐M ESBL‐producing *Escherichia coli* isolates from the Lao People's Democratic Republic, 2004–09. Journal of Antimicrobial Chemotherapy, 67(1), 240–242. 10.1093/jac/dkr434 21987239 PMC3236056

[tbed13558-bib-0081] Suzuki, S. , Shibata, N. , Yamane, K. , Wachino, J. , Ito, K. , & Arakawa, Y. (2009). Change in the prevalence of extended‐spectrum‐beta‐lactamase‐producing *Escherichia coli* in Japan by clonal spread. Journal of Antimicrobial Chemotherapy, 63(1), 72–79. 10.1093/jac/dkn463 19004839

[tbed13558-bib-0082] Tacão, M. , Tavares, R. D. S. , Teixeira, P. , Roxo, I. , Ramalheira, E. , Ferreira, S. , & Henriques, I. (2017). mcr‐1 and blaKPC‐3 in *Escherichia coli* sequence type 744 after meropenem and colistin therapy. Portugal. Emerging Infectious Diseases, 23(8), 1419–1421. 10.3201/eid2308.170162 28726622 PMC5547783

[tbed13558-bib-0083] Tafoukt, R. , Touati, A. , Leangapichart, T. , Bakour, S. , & Rolain, J. M. (2017). Characterization of OXA‐48‐like‐producing Enterobacteriaceae isolated from river water in Algeria. Water Research, 120, 185–189. 10.1016/j.watres.2017.04.073 28486169

[tbed13558-bib-0084] Varela, A. R. , Macedo, G. N. , Nunes, O. C. , & Manaia, C. M. (2015). Genetic characterization of fluoroquinolone resistant *Escherichia coli* from urban streams and municipal and hospital effluents. FEMS Microbiology Ecology, 91(5), fiv015 10.1093/femsec/fiv015 25764463

[tbed13558-bib-0085] Viana, A. L. , Cayô, R. , Avelino, C. C. , Gales, A. C. , Franco, M. C. , & Minarini, L. A. (2013). Extended‐spectrum β‐lactamases in Enterobacteriaceae isolated in Brazil carry distinct types of plasmid‐mediated quinolone resistance genes. Journal of Medical Microbiology, 62(Pt 9), 1326–1331. 10.1099/jmm.0.055970-0 23741024

[tbed13558-bib-0086] Vignoli, R. , Garcia‐Fulgueiras, V. , Cordeiro, N. F. , Bado, I. , Seija, V. , Aguerrebere, P. , … Chabalgoity, J. (2016). Extended‐spectrum beta‐lactamases, transferable quinolone resistance, and virulotyping in extra‐intestinal *E. coli* in Uruguay. Journal of Infection in Developing Countries, 10(1), 43–52. 10.3855/jidc.6918 26829536

[tbed13558-bib-0087] Vingopoulou, E. I. , Siarkou, V. I. , Batzias, G. , Kaltsogianni, F. , Sianou, E. , Tzavaras, I. , … Miriagou, V. (2014). Emergence and maintenance of multidrug‐resistant *Escherichia coli* of canine origin harbouring a blaCMY‐2‐IncI1/ST65 plasmid and topoisomerase mutations. Journal of Antimicrobial Chemotherapy, 69(8), 2076–2080. 10.1093/jac/dku090 24722836

[tbed13558-bib-0088] Vogt, D. , Overesch, G. , Endimiani, A. , Collaud, A. , Thomann, A. , & Perreten, V. (2014). Occurrence and genetic characteristics of third‐generation cephalosporin‐resistant *Escherichia coli* in Swiss retail meat. Microbial Drug Resistance, 20(5), 485–494. 10.1089/mdr.2013.0210 24773305

[tbed13558-bib-0089] Wang, J. , Ma, Z. B. , Zeng, Z. L. , Yang, X. W. , Huang, Y. , & Liu, J. H. (2017). The role of wildlife (wildbirds) in the global transmission of antimicrobial resistance genes. Zoological Research, 38(2), 55–80. 10.24272/j.issn.2095-8137.2017.003 28409502 PMC5396029

[tbed13558-bib-0090] Yamamoto, T. , Takano, T. , Iwao, Y. , & Hishinuma, A. (2011). Emergence of NDM‐1‐positive capsulated *Escherichia coli* with high resistance to serum killing in Japan. Journal of Infection Chemotherapy, 17(3), 435–439. 10.1007/s10156-011-0232-3 21437680

[tbed13558-bib-0091] Zerbino, D. R. , & Birney, E. (2008). Velvet: Algorithms for de novo short read assembly using de Bruijn graphs. Genome Research, 18(5), 821–829. 10.1101/gr.074492.107 18349386 PMC2336801

[tbed13558-bib-0092] Zhao, W. H. , & Hu, Z. Q. (2013). Epidemiology and genetics of CTX‐M extended‐spectrum beta‐lactamases in Gram‐negative bacteria. Critical Reviews in Microbiology, 39(1), 79–101. 10.3109/1040841X.2012.691460 22697133 PMC4086240

[tbed13558-bib-0093] Zurfluh, K. , Nuesch‐Inderbinen, M. , Klumpp, J. , Poirel, L. , Nordmann, P. , & Stephan, R. (2017). Key features of mcr‐1‐bearing plasmids from *Escherichia coli* isolated from humans and food. Antimicrobial Resistance & Infection Control, 6(91), 1–6. 10.1186/s13756-017-0250-8 PMC5585931 28878890

[tbed13558-bib-0094] Zurfluh, K. , Wang, J. , Klumpp, J. , Nuesch‐Inderbinen, M. , Fanning, S. , & Stephan, R. (2014). Vertical transmission of highly similar blaCTX‐M‐1‐harboring IncI1 plasmids in *Escherichia coli* with different MLST types in the poultry production pyramid. Frontiers in Microbiology, 5(519), 1–7. 10.3389/fmicb.2014.00519 25324838 PMC4179741

