# Supplementary material for: International clones of extended‐spectrum β‐lactamase (CTX‐M)‐producing Escherichia coli in peri‐urban wild animals, Brazil
Source: Transbound Emerg Dis. 2020 Apr 21;67(5):1804–15. doi: 10.1111/tbed.13558 (PMC7540485; doi:10.1111/tbed.13558)
Supplement: Supplementary file 1 — Fig S1 [file TBED-67-1804-s001.docx]

**Supporting Information**

**Figure S1.**cgMLST-based phylogenetic tree of CTX-M-positive *E. coli* strains isolated from peri-urban wild animals in Brazil. The phylogenetic tree is based on cgMLST analyses. Visualization was performed using iTOL.
